# Effective implementation of primary school-based healthy lifestyle programmes: a qualitative study of views of school staff

**DOI:** 10.1186/s12889-019-7550-2

**Published:** 2019-09-09

**Authors:** Rhiannon Eleanor Day, Pinki Sahota, Meaghan Sarah Christian

**Affiliations:** School of Clinical and Applied Sciences, Leeds Beckett University, CL615A, City Campus, Leeds, LS1 3HE UK

**Keywords:** Childhood obesity, primary schools, healthy lifestyle programmes, school staff views

## Abstract

**Background:**

Primary schools are valuable settings to implement healthy lifestyle (healthy eating and physical activity) interventions, aimed at targeting childhood obesity. This study explored school staff perceptions of factors that hinder and enable successful implementation and sustainability of healthy lifestyle interventions in primary schools. Qualitative data was pooled and analysed from two evaluations carried out in primary schools in North England: a feasibility study of a nutrition and physical activity educational programme (PhunkyFoods Feasibility Study), and an evaluation of a healthy eating programme (The Food Dudes Evaluation).

**Methods:**

Sixty-five qualitative semi-structured interviews were conducted with head teachers, teachers, catering managers, designated school-based programme coordinators and programme staff supporting schools with programme delivery, at 14 schools involved in both evaluations. Thematic analysis was undertaken and emergent themes categorised using a framework for successful implementation by Durlak and Dupre (2008).

**Results:**

Overall, all schools were delivering a range of healthy lifestyle programmes, often with overlapping content. Perceived challenges to implementation of individual programmes included: limited time, timing of implementation, limited training and support, insufficient resources, capacity and facilities, staff perceptions of intervention and perceived skill-proficiency (for cooking and physical activities). Short-term funding, lack of external and internal support were perceived to hinder sustainability. Staff recommendations for successful implementation of future programmes included: extended training and planning time, sufficient capacity, external support for delivery, good resources (interactive, practical and adaptable), and facilities for cooking, healthy eating, gardening and physical activities. Head teachers need to prioritise delivery of a few key healthy lifestyle programmes, in an overcrowded curriculum. Schools need to employ strategies to engage participation of staff, pupils and parents long term.

**Conclusions:**

Effective implementation of school-based healthy lifestyle programmes was thought to be aided by flexible and adaptable programmes, enabling good contextual fit, well-resourced programmes and effective leadership at multiple levels, pupil (pupils support delivery) and parent involvement. To facilitate sustainability, it was perceived that programmes need to be integrated within the curriculum and school policies long term, with sustained support from head teachers and staff. These findings are relevant to programme developers, policy makers and those involved in delivering interventions.

**Electronic supplementary material:**

The online version of this article (10.1186/s12889-019-7550-2) contains supplementary material, which is available to authorized users.

## Background

Childhood Obesity is a serious public health concern both globally [1] and in the UK [2]. Latest data from the National Child Measurement Programme (NCMP) in England in 2016–17, indicated that almost a quarter of children starting primary school (aged 4–5 years), were already overweight or obese. This increased to over a third of children by the end of primary school (aged 10–11 years) [3]. The long term health and social consequences of childhood obesity are well-established [4], and the adverse health impacts of childhood obesity are likely to continue into adulthood [5–8]. Once established, obesity is difficult to reverse [9], thus strengthening the case for primary prevention [10].

The World Health Organisation (WHO) suggests that to target obesity effectively, action in multiple settings, with a range of stakeholders and a variety of approaches is required [11]. Furthermore, a key component of this is to target the key determinants of obesity (nutrition and physical activity), through complex, multi-component interventions implemented in schools [10]. Research evidence and practice guidelines highlight the critical role of schools in obesity prevention [12–14]. Schools hold much potential for influencing healthy eating and physical activity behaviours, because children have long-term contact and spend much of their waking hours at school [15, 16].

Evidence exploring the effectiveness of single or multi-component school-based interventions, targeting dietary intake and/or physical activity or sedentary behaviour, has produced mixed findings. Some systematic reviews have indicated potential in improving eating habits and physical activity in children at school and at home [10, 17, 18], as well as decreasing sedentary behaviour [19]. Other systematic reviews however, have indicated limited effectiveness on physical activity [20], nutrition behaviour [21, 22], and reducing sedentary behaviour and BMI [19, 23, 24]. The variability between studies indicates both the complexity of these interventions and their evaluations, and the challenges in identifying key successful components. Also highlighting the importance of understanding why only some interventions were effective [7].

In order to determine the effectiveness and sustainability of healthy lifestyle interventions, we need to understand which specific components of the intervention determine behaviour change [25], as well as the context, how and to what extent interventions are implemented in real-world settings [26]. Transferring effective programmes into real-world settings and maintaining them there is a long-term complex process, which requires dealing with complex phases of programme diffusion (the spread of evidence based promotion, prevention or treatment programmes) [26]. These phases include the following: (1) dissemination, how well information about a programme’s existence and value is supplied to communities; (2) adoption, whether a local organisation or group decides to try out the programme; (3) implementation, how well the programme is conducted during a trial period, and (4) sustainability, whether the programme is maintained over time [26]. For programmes to be successful and people to benefit, diffusion must be successful in multiple communities, at each stage of the process, from dissemination through to sustainability [26]. Durlak and Dupre [26], suggest that in order to bridge the gap between programme development and adoption of effective health promotion interventions on a scale large enough to induce population level health changes, there a critical need to understand the factors related to programme implementation. Process evaluations of implementation, can help identify how to implement interventions, what works, for whom, in which contexts and why [7], however these are rarely conducted [26]. It is therefore critical to improve understanding of the factors that support effective implementation of school-based health promotion programmes [26], but these studies are lacking [27]. Understanding the factors that influence adoption and implementation of health programmes in school-based settings is challenging, due to the belief that schools have constantly shifting broader contexts [27]. There is a dearth of research, focussing on methods or strategies used to enhance implementation, sustainability and scale-up of dietary and/or physical activity interventions, conducted in the primary school setting [27, 28]. Many existing studies have only included small samples, without the perspectives of a large variety of different school staff involved in programme delivery. There is particularly a lack of school-based studies carried out in the UK, exploring the views of school staff around facilitators and barriers to successful healthy eating and physical activity programme implementation and sustainability [7], as well as recommendations to overcome identified barriers, to inform future healthy lifestyle programmes.

Previous systematic narrative reviews have outlined frameworks of factors needed for effective implementation of health promotion and prevention programmes in health services [29, 30] and in schools (mainly mental health programmes) [26, 31–33]. Frameworks of factors for successful implementation [26, 29, 31, 33], have proposed constructs influencing effective implementation at community level, organisation level (for example school level), intervention level and individual level (implementers). Durlak and Dupre [26], conducted a systematic review of more than 500 health promotion interventions (including physical health interventions), targeting children and youth, and identified 23 contextual factors that they fit into a multi-level framework outlining effective implementation. Their framework outlined that the implementation process is influenced by variables present in five categories: innovations, providers, communities and aspects of the prevention delivery system (such as organisational capacity), and the prevention support system (training and technical assistance). Durlak and Dupre [26], hypothesise that when variables in all five categories interact under favourable circumstances, this leads to effective implementation, that is a process for implementing the intervention as planned [26]. This framework underpins the discussion of factors that influence implementation in our study.

Our study explores the perspectives of a range of school stakeholders (head teachers, catering managers, teachers, curriculum coordinators, assigned programme coordinators, programme provider team), relating to factors facilitating and hindering successful implementation and sustainability of healthy eating and physical activity programmes in the primary school setting. Their recommendations for overcoming identified barriers, and for effective implementation of future healthy lifestyle programmes are also presented. These findings can help guide future planning and provision for healthy lifestyle programmes delivered in the school environment.

## Methods

### Data

Qualitative interview data was pooled from all intervention and control schools, participating in two separate evaluations of healthy lifestyle programmes, within primary schools in the North of England. The first evaluation was an 18-month feasibility study (PhunkyFoods Feasibility study - PFS), testing the acceptability and feasibility of the PhunkyFoods Programme (November 2012 to July 2014). The PhunkyFoods programme is an early years and primary school behaviour change programme for children aged 5–11 years. It is a programme of healthy lifestyle activities, lesson plans and resources, based on the Health Promoting Schools approach. It aims to educate schools, pupils and the wider school community to improve healthy eating and physical activity knowledge and behaviours [34]. The programme team provide teaching staff with training and learning resources to deliver healthy lifestyle activities and lessons. Schools were recruited for the PFS by inviting all primary schools within a town in the North of England, except independent and special schools, and schools with only Key Stage 2 pupils (aged 7–11 years) to participate. This was achieved via letters and information sheets, with follow up visits to schools that showed initial interest. From a sample of 70 primary schools, eight primary schools (4 intervention and 4 control) were recruited and the head teachers provided consent to participate.

The second evaluation was of a healthy eating programme called the Food Dudes Programme (FDE) (April 2015–December 2016) [35]. The Food Dudes programme is a healthy eating intervention for children aged 5–11 years. It was designed by psychologists, using behavioural principles and theory. It aims to increase children’s fruit and vegetable intake at school and at home, using role modelling (through programme characters), repeated tasting, recording of fruit and vegetable consumption and rewards to promote palate adaptation and long-term behaviour change. A behaviour change specialist provided training and support for staff delivering the programme. Schools were recruited by obtaining a list of primary schools in a Local Clinical Commissioning Group in a city in the North of England. These schools were recruited via phone calls, emails and provided with information sheets. Head teachers gave consent to participate. Six schools (3 intervention and 3 control) participated.

All schools involved in both evaluations also delivered a variety of additional healthy eating and physical activity programmes and initiatives. Descriptions of all healthy lifestyle programmes delivered at the primary schools, including full descriptions of the Food Dudes and the PhunkyFoods programmes are included as Additional file 1.

### Recruitment of study participants for the current study

A purposive sample of school staff, were invited to participate in an interview at the 8 schools involved in the PFS and at 6 schools involved in the FDE. They comprised of headteachers, catering managers, designated school-based programme coordinators and teaching staff. Year 2 (aged 6–7 years) and Year 4 (aged 8–9 years) teaching staff, were invited to be interviewed for the PFS, and Year 2 and Year 3 (aged 7–8 years) teaching staff, were invited to be interviewed for the FDE. These were the teaching staff of the year groups involved in the evaluations. Members of the programme delivery team, assigned to support schools with delivery of the PhunkyFoods programme (community support worker) and the Food Dudes programme (behaviour change specialist), were also invited to be interviewed. Information sheets and consent forms were given to all potential participants requesting them to contact the research team to organise an interview if they wished to participate.

### Data collection

For the PFS, 54 face-to-face semi-structured interviews were carried out at two time periods in the 8 primary schools. The first, approximately 6 months post implementation of the PhunkyFoods programme (at the end of the first academic year), to ascertain factors related to implementation, and then at approximately 18 months post implementation (at the end of the second academic year), to ascertain factors related to sustainability.

The interviews aimed to explore the healthy lifestyle programmes and initiatives delivered at the school, roles within programmes, training and support, perceived effectiveness, challenges and barriers to success. Questions also addressed key learning from implementation of programmes, sustainability and general recommendations for healthy eating and physical activity school-based programmes. The topic guides are included as Additional file 2. The topic guide was adapted depending on the role and knowledge related to programme delivery, of the interviewee. The interview schedules were developed and piloted with one headteacher and one catering manager at an unrelated primary school. Interviews lasting approximately 20–40 min, were conducted within school by two female researchers, RED (Master of Public Health) and MSC (PhD), during school time. RED and MSC have substantial training and experience in qualitative research methods. The same interview questions were used with school staff at both time points.

For the FDE, similar interview questions were used with school staff at all schools. Ten semi-structured interviews were carried out at 5 schools, approximately 8 months following programme implementation (at the end of the academic year), to ascertain factors relating to implementation and sustainability. One interview was carried out at the sixth school, approximately 1 month following implementation, as this school had received the programme later than the others, and data needed to be captured during the study period. Interviews lasting approximately 20–50 min, were conducted within school by one researcher (RED) during school time, except one interview that was conducted over the telephone.

All participants within both evaluations provided written informed consent to be interviewed and digitally recorded. No-one else was present during the interviews besides the participants and researchers. The researchers conducting the interviews had made contact with the head teachers at the schools during the recruitment period, therefore were known to them prior to interview. The researchers had already worked with Year 2 and Year 4 teaching staff at the 8 PFS schools, during previous data collection with pupils, therefore were known to these specific teachers. The researchers were not known to all other staff participating. Additional field notes were made both during and immediately following interviews. Data saturation was considered in relation to the data provided by an individual participant, i.e. was achieved at a particular point within a specific interview when the researcher felt that they had gained a full understanding of the participants’ perspectives on each topic area [36].

### Ethical approval

Ethical approval for both studies was provided by Leeds Beckett University, Faculty of Health and Social Sciences Ethics Review Committee.

### Data analysis

All recordings of interviews were listened to for familiarisation and transcribed using a process of iterative listening, whereby only key passages were transcribed verbatim. It was felt that this form of partial transcription would suit the type of analysis needed for the evaluations [37]. This was carried out within 7 days post interview. Any additional relevant information from field notes was added to transcripts. Transcripts were not returned to school staff to comment on, nor were participants asked to provide feedback on the findings, due to limited staff time for participation in the study. Qualitative interview data was pooled from all schools in both evaluations (*N* = 14). A thematic analysis of data was carried out using both an inductive and deductive approach [38]. Transcripts were read and re-read for familiarisation. Coding was carried out manually using highlighters to indicate potential patterns. An initial set of codes was developed by RED, verified by MSC and applied to the data. Some codes were identified a priori, using the interview topic guide and the implementation framework used in the analysis, while others emerged inductively from the data. Coded data was categorised and collated into themes, using tables in Microsoft Word 2016, with all the relevant coded extracts collated within identified overarching themes and sub-themes. The themes generated were reviewed and refined, and discussed between members of the study team for consensus validation. The emergent themes relating to factors hindering and facilitating programme implementation, were categorised using the implementation framework outlined by Durlak and Dupre [26]. The Consolidated Criteria for reporting Qualitative Studies (COREQ-32 item checklist) was applied in the report of findings [39].

## Results

### Description of interview participants

A breakdown of participants is presented in Table 1. A number of additional members of teaching staff, who were not originally invited, also agreed to participate in an interview. Twenty members of staff were interviewed at both time points of data collection for the PFS (6 catering managers, 4 head teachers, 4 PhunkyFoods programme coordinators, 6 teachers). For the FDE, only a small number of staff agreed to be interviewed in total, as many staff reported having no time to take part. No Year 2 and Year 3 teaching staff could find the time to be interviewed. One interview included three participants for the purpose of convenience, as staff were short of time.

**Table 1:** Description of interview participants

	PFS	FDE	Total
Total number of participants invited	36	28	64
Total number of interviews	54^a^	11^b^	65
Number of interviews by role			
Catering managers	12	5	17
Head teachers	8	3	11
Year 1 teacher^c^	1	–	1
Year 2 teacher	11	–	11
Year 4 teacher	10	–	10
Year 6 teacher^c^	1	–	1
Reception teacher (and HSC)^c^	1	–	1
Assigned programme coordinators^d^	7	2	9
PhunkyClub coordinator^d^	2	–	2
PSHE coordinator^c^ (Year 5 teacher)	–	1	1
Food Technology coordinator^c^	–	1	1
Community support worker	1	–	1
Behaviour change specialist	–	1	1

*HSC* Healthy school coordinator

^a^Interviews were conducted at two time periods (6 months and 18 months post implementation of the PhunkyFoods programme)

^b^One interview had three participants: head teacher, programme coordinator and behaviour change specialist

^c^Additional staff took part in an interview who were not originally invited

^d^Members of staff assigned to coordinate the PhunkyFoods programme: head teacher, teacher, nutrition action manager, PSHE (Personal, Social and Health Education) coordinator. PhunkyClub coordinator (after school PhunkyFoods club): teaching assistant. Members of staff assigned to coordinate the Food Dudes programme: Year 3 teacher, school bursar

### Factors hindering and facilitating successful implementation and sustainability of healthy lifestyle programmes

A number of factors perceived to challenge and facilitate successful implementation and sustainability of healthy lifestyle programmes, were identified by school stakeholders. Table 2 presents the perceived barriers to implementation and sustainability identified by staff, and Table 3 presents the perceived facilitators, with illustrative quotes from participants. Roman numerals (superscript) have been used to demonstrate how the themes align with the categories of factors identified for effective implementation in the implementation framework by Durlak and Dupre [26]. These factors include: I community level factors, i.e. funding; II provider characteristics, i.e. perceptions of need for and benefit of innovation and skill proficiency; III characteristics of the innovation, i.e. adaptability (flexibility) and compatibility (contextual appropriateness), availability and quality of programme activity resources; IV factors relevant to the prevention delivery System (factors related to organisational capacity), i.e. integration of new programmes, shared decision making (local input, community participation and collaboration), shared vision (commitment and staff buy-in), formulation of tasks (teams, effective human resource management), availability and quality of resources such as personnel and facilities, and leadership and programme champion; and V factors related to the prevention support system, i.e. training and technical support.

**Table 2:** Perceived factors hindering implementation and sustainability of healthy lifestyle programmes at the primary schools

	Illustrative quotations
Factors hindering implementation	
Time constraints^IV^	“We get asked to do a lot of things, just the opportunity to do them when we have to focus on driving progress and attainment as a priority” (Programme coordinator, school 9, FDE)
	“The food tasting was supposed to be 10 minutes but that was one of the challenges in that it impacted quite heavily on lesson time in the morning …” (Head teacher, school 9, FDE)
	“We didn’t do a fruit platter today because we didn’t have time … we were coming in early to get all the fruit prepared (for the programme), as well as the usual meals and salads” (Catering manager, school 11, FDE)
Timing of implementation^a^	“… I think because we got it (PhunkyFoods programme) in the middle of the year, it was hard to implement it. It would have been better to have it now to look at it, ready for September” (Programme coordinator, school 1, PFS)
Training and technical support^V^	“… I mean it would just be different for us if we had more training on it, if we were more aware of it, because obviously time wise when you’re planning lessons, you don’t have hours to kind of sit and go through things” (Year 1 teacher, school 1, PFS)
	“We tried to get them in (programme support team), and then they said they would come in, and then it was time of when they could come in, and who was going to be here to let them in. So then we waited and waited and in the end the teaching assistant and I did it” (Year 2 teacher, school 2, PFS)
Availability and quality of resources (personnel and facilities)^IV^	“Lots of children wanted to do it (cooking club), but we could only choose 12 children due to limited staffing” (Programme coordinator, school 11, FDE)
	“We were involved with Focus on Food. They provide a cooking bus which, children go onto the bus and cook. We were meant to be one of the school’s to use it, but couldn’t get the bus up to our school” (Reception teacher, school 6, PFS)
	“We would like to start a cooking club but we don’t have the space” (Head teacher, school 2, PFS). “We don’t have many after school activities due to space restrictions” (Year 2 teacher, school 2, PFS)
Funding^I^	“Cost is a huge issue around delivering it, we need funding to deliver them” (Head teacher, school 12, FDE)
Teacher characteristics (engagement, perceived need for and benefit of innovation and skill proficiency)^II^	“Well I think initially, you know, obviously like any new initiative, the staff are probably a little bit concerned that it’s another job that’s been added on top of already what they’re doing” (Head teacher, school 2, PFS)
	“… with the Phunkyfoods thing, there are so many kind of food initiatives out there, I’m using some of the Food for Life things as well because that was more appropriate” (Year 1 teacher, school 1, PFS)
	“We haven’t really looked at the physical ones (programme resources) because we tend to have quite good PE curriculum” (Programme coordinator, school 1, PFS)
	“… No specific training for staff to deliver the sports programmes. Staff are just expected to run these programmes in school” (Year 4 teacher, school 5, PFS)
	“There might be some training implications with current staff needing training to deliver new cooking activities in the curriculum” (Head teacher, school 6, PFS)
Effective leadership^IV^	“… the lead role went on maternity leave and there’s an awful lot of change going on at the moment and it’s (the PhunkyFoods programme) taken more of a side line” (Year 4 teacher, school 5, PFS)
	“It’s kind of because it’s not statutory we’ve not been told you need to use this for anything, it’s kind of dip in when you want, maybe there’s some things there, I mean we don’t have to use it, so it’s left up to us” (Year 1 teacher, school 1, PFS)
Parent participation and support^IV^	“When we put on workshops, parent participation is poor, we have tried incentives but it is still limited” (Head teacher, school 8, PFS)
	“They (parents) wanted their pack lunch policy to suit their children’s likes and dislikes, it isn’t necessarily in line with what we would like … it’s been very hard” (Food technology coordinator, school 13, FDE)
	“The more vulnerable children have parents that are not able to let their children attend sports clubs for socioeconomic reasons” (Head teacher, school 7, PFS)
Factors hindering sustainability	
Funding	“I was ordering fruit (for the programme), that was just too expensive to continue. I’ve tried to cut back on that, it’s like your strawberries, your blueberries, your blackberries, my manager was like you need to get your stock down” (Catering manager, school 11, FDE)
Staff capacity and support	“We have stopped the PhunkyFoods club this term because they do the drama club now instead. There was only one teacher available to run it” (Programme coordinator, school 1, PFS)
	“… if we had more staff, it (cooking club) could be more of a regular thing” (Programme coordinator, school 11 FDE)

Superscript roman numerals refer to categories within the Durlak and Dupre model of factors affecting implementation [26]:

^I^ Community Level Factors

^II^ Provider Characteristics

^III^ Characteristics of the Innovation

^IV^ Factors Relevant to the Prevention Delivery System: Organisational Capacity

^V^ Factors Related to the Prevention Support System

^a^ Factors influencing implementation not identified in the model by Durlak and Dupre [26]

**Table 3:** Perceived factors facilitating implementation and sustainability of healthy lifestyle programmes at the primary schools

	Illustrative quotations
Factors facilitating implementation	
Contextual appropriateness and adaptability^III^	“Every school is different, this is what works for one school, this is what works for another school, and until you find you’re actually there in that specific school, how we work out (our programme), this will work better for us” (Behaviour change specialist, FDE)
	“… I felt like I was jumping over hurdles for the sake of a certificate (for the Food for Life Programme). It has been trimmed down now and is more appropriate for the school” (Head teacher, school 6, PFS)
	“We did it (The Food Dudes programme) a little bit differently. We continued a scheme at playtime where children would tick off their level cards when they had fruit. We gave rewards out in the classroom, as all the children have their school dinners at different times, so we couldn’t do it in the dining room” (Programme coordinator, school 11, FDE)
	*“*We aren’t delivering it in the here’s a lesson, we deliver it every week, because it needs to fit with what’s right for the children in the school … and in addition to this, we’ve then done it as a club” (Head teacher, school 1, PFS)
	“I left it very much to the individual classes to run it how they felt, which actually worked better I think for them” (Programme coordinator, school 11, FDE)
Availability and quality of resources (personnel and facilities)^IV^	“We need staff capacity to run programmes, like the healthy schools award” (Programme coordinator, school 9, FDE)
Availability and quality of programme activity resources^III^	“We could probably do with some more (resources), a lot of it is DIY and maybe some sort of scheme to run to … a bit of guidance on which way it goes” (Year 4 teacher, school 8, PFS)
	“In terms of delivery, I think the more hands on activities the children can do, like first-hand experiences the better” (Year 2 teacher, school 3, PFS)
Integration of new programmes (in the curriculum, school structures and food policies) ^III and IV^	“Keeping it within the curriculum, make it integral not a bolt on or after school club and everyone gets it. Keep it within the curriculum so everyone receives it” (Year 2 teacher, school 7, PFS)
Teacher characteristics (perceived need for and benefit of innovation)^II^	“… where they (staff) can see a relevant link to what the children are learning, I think they’re more positive about it and the staff are very good at taking things on and running with it really” (Head teacher, school 2, PFS)
Shared vision (commitment and staff buy-in), leadership, programme coordinator (champion) and managerial/ administrative support^IV^	“It (the Food Dudes programme) was successful because we have a strong team of senior and non-teaching staff coordinating the programme” (Programme coordinator, school 9, FDE)
	“The head gets involved as much as possible and introduced ‘meat free Mondays’” (Catering manager, school 10, FDE)
	“… and that (success) was the staff encouraging it … our ultimate, is to make sure those children have a healthy diet when they are with us … there was a buzz around it (programme), support around it, excitement around it and a lot of focus on it” (Programme coordinator, school 9, FDE)
	“We’ve had lots of staff meetings about it, I know XX (programme coordinator) has been kind of the drive behind the initiative. She’s done really well with showing us and demonstrating all the resources” (Year 2 teacher, school 5, PFS)
Training and technical support^V^	“I think doing a hands on training and giving people the time in a training session to go away and sort of plan it, yeah and just some dedicated time” (programme coordinator, school 1, PFS)
	“… those contacts and that ability for people to do those key aspects is very important as part of the programme … sometimes having somebody to contact and say look I want you to do this, can you put me in contact with, that is a very important element” (Programme coordinator, school 3, PFS)
	“You’ve got to be wary, yes you want schools to engage with you, with the programme, but also they’re going to have priorities that you know, you’ve got to be careful to get the balance right, so you are not seen as invasive” (Programme provider, PFS)
Formulation of tasks (teams, effective human resource management)^IV^	“… it was about finding a member of staff who could supervise them (pupils) … I think that’s quite an important thing to make sure there is a consistent member of staff who can do that” (Head teacher, school 9, FDE)
	“We have thought about using specialist teachers, with more time to deliver them (programmes) and who might be passionate about delivering healthy lifestyle messages” (Head teacher, school 10, FDE)
	“I need another member of staff, giving me somebody from half past nine, to take the pressure off me” (Catering manager, school 9, FDE)
Parent and community participation (shared decision making)^IV^	“… we need parents to understand how to work with fruit and veg, knowledge of fruit and veg, do cooking (with them), we need to focus on the parents” (Programme coordinator, school 9, FDE)
	“Parents are on the school nutrition action committee group, so parents have been spoken to about healthy eating in school, they’ve been surveyed” (Head teacher, school 1, PFS)
	“… within that garden area, there’s allotments and we try and encourage parents and the community to come and grow fruit and veg and they can take that produce away with them … the children are also involved in that” (Head teacher, school 8, PFS).
	“… we had highlighted we needed support workers to help embed the programme in local communities” (Community support worker, PFS)
Pupil characteristics, engagement and motivation^a^	“We have food ambassadors working in the hall, giving other children stickers for healthy food behaviours” (Head teacher, school 10, FDE)
	“Year 6 did a lot for us, they were monitoring the cards (level cards) and monitoring the prizes, because we didn’t have time for that” (Catering manager, school, 12, FDE
Factors facilitating sustainability	
Sustained engagement in programmes and integration long-term	“In order for it to be sustained it has to keep coming round because if parents don’t buy things at home, then children lose the taste for it and they go back to not liking it and everything else” (Head teacher, school 12, FDE)
	“… I think the most important thing is making it sustainable because if you have a big push to begin with and it wanes, then the impact is going to be much less. I think it’s important when we’re weaving it in, it becomes a sustainable part of what we do.” (Head teacher, school 2, PFS)
	“I think more time needs to be spent over it, extended rather than focused in one week for example” (Reception teacher, school 6, PFS)
Head teacher commitment	“… all programmes are sustainable because I am interested in leading on them and passing them onto other people. I will make them sustainable at the school” (Head teacher, school 10, FDE)
Communication about programmes	“We need to reflect on these things at different part of the year, make sure we have the skills in, people share what’s worked well” (Head teacher, school 4, PFS)
	“Communication within staff talking about things really (is important for sustaining programmes)” (Year 4 teacher, school 3, PFS)

Superscript roman numerals refer to categories within the Durlak and Dupre model of factors affecting implementation [26]:

^I^ Community Level Factors

^II^ Provider Characteristics

^III^ Characteristics of the Innovation

^IV^ Factors Relevant to the Prevention Delivery System: Organisational Capacity

^V^ Factors Related to the Prevention Support System

^a^ Factors influencing implementation not identified in the model by Durlak and Dupre [26]

### Factors hindering implementation of healthy lifestyle programmes

#### Time constraints (IV prevention delivery system: organisational capacity)

There was a general consensus among all staff members that having limited time was perceived to be the main challenge to implementing new healthy lifestyle programmes. Competing priorities and an already congested curriculum, meant that head teachers struggled to prioritise healthy lifestyle teaching and programmes generally. Healthy lifestyle teaching occasionally had to be delivered in assembly time, rather than lesson time, due to lack of time in the curriculum.

The schools were delivering a large range of healthy eating and physical activity programmes (in lesson time and after school). Some of these programmes had overlapping content, particularly those educating around healthy food, food preparation, food waste and food sustainability (for example, Healthy School award, Food for Life partnership award, PhunkyFoods programme, Tesco Eat Happy project, Fuel for School programme and cooking clubs). These programmes compete for curriculum time, when the curriculum is already overcrowded. Implementation and acceptance of new programmes, could be hindered by over-burdening staff with too many programmes. Having sufficient time therefore, to deliver programme activities, particularly those that were not already integrated into lesson plans (in addition to the usual prescribed curriculum), was perceived to be a recurrent challenge. Catering staff also struggled to find time for additional food preparation, such as fruit and vegetable preparation for tasting activities and creative fruit displays for lunchtimes, without additional staff support and time. Although time constraints were not included as a specific factor influencing implementation in the model by Durlak and Dupre [26], the factors influencing time constraints in our study (competing priorities with a heavily congested curriculum, time to prepare and deliver programmes, and teacher overload), might reflect a resourcing issue at school level. This has thus been classified as a factor relating to the prevention delivery system [26].

#### Timing of programme implementation

Poor timing of healthy lifestyle programme implementation, such as receiving the programme half way through the academic year (when curriculum planning was already completed for the year), was another perceived important barrier to adoption and implementation of programmes. Timing of programme implementation was not specifically included as a factor influencing implementation in the Durlak and Dupre model [26].

#### Training and technical support (V prevention support system)

Insufficient training was also perceived to be an important barrier to healthy eating and physical activity programme implementation. Teaching staff that were not given adequate training and preparation time to review the programme resources before implementation, were less accepting of new programmes, preferred to use more familiar programme resources and took less ownership over aspects of programmes. Furthermore, new staff appointed after a programme had started, were sometimes insufficiently trained or not given the resources to adopt a programme. A lack of sufficient communication and technical support from the programme team (providing the programme) towards schools, was also perceived to hinder implementation. For example, one school experienced difficulties with accessing timely support from the programme team to install ground beds in the garden for a growing scheme.

#### Availability and quality of resources (personnel and facilities) (IV prevention delivery system: organisational capacity)

Another important barrier to implementation discussed by staff was lack of adequate resources and facilities (particularly for physical activities, healthy eating education and cooking activities). Limited staff capacity to deliver after school healthy lifestyle programmes (for example cooking clubs), restricted the frequency and sustainability of delivery and number of children who could attend. Inadequate infrastructure, equipment and school space, prevented implementation of school cooking, gardening activities and sports clubs particularly. Adequate school resources and facilities to implement programmes, were thought to relate to organisational capacity, and have therefore been classified as factors relating to the prevention delivery system, from the Durlak and Dupre model [26].

#### Funding (I community level)

Funding was a recurrent challenge for schools. Limited funding or uncertainty about future funding, had a negative impact on access to certain healthy lifestyle programmes, such as the Food for Life or Healthy Schools programmes.

#### Teacher characteristics (engagement, perceived need for and benefit of innovation and skill proficiency) (II provider characteristics)

Where staff were using programmes that were perceived to be familiar and already well-established in the curriculum, such as the Physical Education curriculum, staff failed to see a need for new similar programmes, with the same key targets for behaviour change. There was therefore there some resistance to wanting to adopt these programmes initially. Fear of additional workload for staff, would also occasionally hinder programme acceptance and thus implementation initially. Lack of sufficient training and under-skilled teaching staff, was also a perceived barrier to effective implementation of physical activity or cooking activities particularly.

#### Effective leadership (IV prevention delivery system: organisational capacity)

Implementation of programmes was also often dependent on effective leadership from an in house programme coordinator (a designated member of staff instructing on programme delivery and facilitating access to training and resources). Lack of effective and sustained leadership from a programme coordinator, would often result in programmes not being prioritised and staff not being encouraged to use programme resources. Furthermore, little guidance and communication from programme coordinators around how a programme should be delivered, would cause large variability in level of programme delivery between teaching staff, potentially compromising programme fidelity.

#### Parent participation and support (IV prevention delivery system: organisational capacity)

Effective engagement of parents was universally acknowledged to be one of the most challenging and least successful elements of healthy lifestyle programme implementation. Parent attendance at meetings, activities and events in school, was generally reported to be low. Parent attitude and limited time were considered to be the main reasons. Some parents were reported to be less compliant with school food policies, packed lunch policies and healthy snack provision. Equally engaging disadvantaged pupils and parents, in school food programmes and after school sports activities, was also perceived to be challenging. There was also a perceived barrier to engaging pupils (and parents), in after school clubs, when for example they needed to attend religious studies or prayer time after school. Although not specifically included within the model by Durlak and Dupre [26], it was thought that parent involvement could be categorised alongside community involvement within ‘shared decision making’ in the model, and has therefore been classified as a factor relating to the prevention delivery system.

#### Factors hindering sustainability

Securing sustainable funding was perceived to be a critical barrier to long-term sustainability of nutrition and physical activity programmes. Unsustainable external support from Physical Education specialists (due to limited funding), hindered the continued delivery of physical activity programmes at schools. Having sufficient staff capacity and staff support to secure continuation of programmes at the schools, was considered to be another significant challenge, particularly for after school cooking and physical activity clubs.

### Factors facilitating implementation of healthy lifestyle programmes

#### Contextual appropriateness and adaptability (III characteristics of the innovation)

Adaptations to programme delivery to suit the school context (timings, locations, resources, format, for example after school club or within the curriculum), were seen to facilitate more successful implementation. Programme coordinators and teaching staff, felt it important to have flexibility and autonomy over how teaching staff delivered programmes within their classrooms (with localised decision-making), as this was seen to increase likelihood of programme adoption through acceptance and ownership.

#### Availability and quality of resources (personnel and facilities) (IV prevention delivery system: organisational capacity)

Sufficient staff capacity, resources and adequate facilities for cooking, gardening and physical activities, were considered critical for effective implementation of healthy lifestyle programmes.

#### Availability and quality of programme activity resources (III characteristics of the innovation)

Well-resourced programmes, with a variety of engaging, interactive and “hands on” resources were seen to facilitate delivery and increase programme acceptance. Moreover, using a variety of resources from different programmes, was thought to be beneficial by some staff. Incentives (such as rewards, stickers, certificates of achievement) that encouraged children to try new fruits and vegetables and to bring in healthy lunchboxes, were perceived to be enable more successful implementation and were perceived to improve healthy eating behaviours. The use of programme characters or role models (especially on the DVDs), were perceived to help engage pupils and drive interest in learning about healthy lifestyle. The availability of good quality programme activity resources (provided by the programme), was thought to relate to compatibility of the intervention, and was therefore classified as a factor relating to characteristics of the innovation, from the Durlak and Dupre model [26].

#### Integration of new programmes (in the curriculum, school structures and food policies) (IV prevention delivery system: organisational capacity and III characteristics of the innovation)

It was considered that healthy lifestyle programmes need to be integrated within the curriculum, school structure or school food policies, to ensure prioritisation and encourage implementation. This would allow teachers to incorporate programme resources and activities within their teaching plans, so that they do not have to deliver a programme in addition to their usual curriculum. Furthermore, delivering healthy eating programmes in a cross-curricular manner in a range of subject areas, rather than as a singular unit, was recommended for successful integration within the school curricula. The effective integration of new programmes was thought to be a factor relating to both organisational capacity (the extent to which the school can incorporate it into its existing practices and routines), as well as contextual appropriateness (how it fits with the school’s priorities and values) and adaptability of the intervention (to fit the schools preferences and practices). It has thus been classified as a factor relating to both the prevention delivery system and characteristics of the innovation, from the Durlak and Dupre model [26].

#### Teacher characteristics (perceived need for and benefit of innovation) (II provider characteristics)

Also considered important by staff was to ensure that teaching staff were confident on how new programme goals aligned with curriculum objectives. Staff that understood the value of new healthy lifestyle programmes, in enhancing children’s learning and contributing to academic achievement, were then more likely to accept and implement them.

#### Shared vision (commitment and staff buy-in), leadership, programme coordinator (champion) and managerial/administrative support (IV prevention delivery system: organisational capacity)

Whole-school involvement, with engagement and collaboration of all school partners (head teacher, teaching staff, catering staff, pupils and parents), were also considered key factors to successful implementation. Effective leadership from the school senior administrative team was perceived to be important to drive programmes forward and facilitate success. It was perceived that head teachers needed to champion programmes and bolster enthusiasm and support from all school staff. Furthermore, effective guidance and leadership from a designated programme coordinator, was seen to be equally important for effective implementation, through engaging staff and keeping focus on a programme.

#### Training and technical support (V prevention support system)

Adequate training, communication and support from programme providers to schools, were also perceived to be important enablers. It was important that staff felt confident and capable, to deliver healthy eating and physical activity messages adequately. Having access to sustained external support for healthy eating and physical activity programmes, was thought to ease implementation and sustainability of programmes. For example nutritionists to deliver healthy eating/cooking teaching, to support the new curriculum that included a focus on diet and cooking, and more support for catering staff preparing foods in the kitchen for food tasting activities. The importance of getting the right balance with support from the programme team supporting delivery in schools, was acknowledged, highlighting the need to provide a sufficient level of support, without being too invasive.

#### Formulation of tasks (teams, effective human resource management) (IV prevention delivery system: organisational capacity)

Ensuring adequate role delineation for delivery, with a sufficient number of suitable staff available to lead on aspects of delivery, was also thought to be a facilitator. For example, allocating staff to supervise healthy eating programme activities with pupils in the school dining room. Establishing “specialist teachers” to deliver all teaching relating to healthy lifestyle, was recommended to ease the burden on other teaching staff, who have not the time to prioritise healthy lifestyle teaching.

#### Parent and community participation (shared decision-making) (IV prevention delivery system: organisational capacity)

Working in partnership with parents on healthy initiatives was considered important to successful implementation, particularly in relation to the provision of healthier packed lunches and encouraging school meals. Involvement with the local community was also thought to be a strong programme attribute. Initiatives such as: community allotments, market stalls selling fruit and vegetables (past sell by date), for the Fuel for School Programme, school shop selling meals to the community, and school meals for local pensioners, were thought to have been successful for fostering community engagement in healthy eating initiatives. Furthermore, the importance of embedding programmes in the local community was highlighted, although it was acknowledged that schools would need additional staff support with this.

#### Pupil characteristics, engagement and motivation

Programmes and initiatives were deemed most successful when pupils were given a central role in delivery. It was reported that pupils who were given leadership roles in the Food Dudes programme (coordinating the completion of level cards and receipt of rewards), valued the responsibility and encouraged their peers to participate. The Food Ambassador programme and School Nutrition Action Group/ School Food Council at several schools, gave pupils a voice over school food policies and healthy eating initiatives, and pupils modelled healthy eating behaviours to their peers. They were considered important strategies for successfully engaging other pupils and fostering ownership. Pupils leading on aspects of programme delivery was not specifically discussed in the Durlak and Dupre model [26].

#### Factors facilitating sustainability

Continuous engagement in healthy lifestyle programmes and initiatives, was considered critical to successful sustainability of programmes. It was considered that programmes need to be sufficiently long in duration to be effective in changing pupil behaviours and need to be therefore institutionally embedded. Furthermore, regular communication about programmes (assemblies, meetings) between staff and between staff and pupils and reflecting on programme delivery, was considered important to encourage staff and pupil engagement. Developing the expertise to deliver programmes in house, was viewed as equally important for sustainability, to foster autonomy and programme ownership. Sustained commitment and support from head teachers and the senior leadership team towards programmes, was considered important to sustain staff engagement and build capacity. For example, establishing a team of dedicated staff to deliver aspects of programmes long term, for example cooking and gardening clubs after school. Whether ongoing funding was secured was also considered critical for programme sustainability.

### Recommendations for overcoming barriers and effective future implementation of healthy lifestyle programmes

#### Training and support

An important recommendation was to provide all members of staff with adequate training and planning time, to review programme resources and incorporate resources into lesson plans. Training and support should be provided through training workshops (whole-school and one-to-one where necessary), visits, phone calls, emails. Training needs to be interactive and practical, with written materials and sharing of success stories between schools. Staff also need to be given sufficient time to pass on training messages to other members of staff that cannot attend, or new staff. If a programme is to be implemented in September at the start of the next academic year, it was recommended that staff receive the training and resources in May of the previous academic year.

#### Resources

Staff also recommended easily accessible programme resources, that can be easily adapted, such as online resources. These were thought to save staff time and could be made more appropriate for pupils with different learning abilities. Interactive resources, relating to cooking and growing foods, online activities on tablets (for example interactive quizzes at the end of topics with certificates for completion), online videos and DVDs for pupils of all ages were also recommended. Also considered important were: physical activity resources for after school clubs; programmes that offer sports that appeal to girls, such as girls only football teams; resources with clear and simple learning objectives, cross-referenced to the national curriculum; and more group work resources. Furthermore, it was perceived that growing resources need to factor in time to grow vegetables, and programmes need more age appropriate resources (videos), and culturally appropriate programme messages. For more effective implementation of healthy eating initiatives, there were recommendations for more volunteers to deliver cooking activities after school, improved cooking facilities, and pre-prepared fruit and vegetables for food tasting initiatives, so that catering staff could spend their time on the presentation of fruit and vegetable displays. Furthermore, sufficient storage facilities for fresh produce, would allow programmes such as the Food Dudes and Fuel for School programme to be more easily implemented.

#### Parent participation and support

Recommended strategies for engaging parents more effectively in programmes included: inviting parents in for school meal taster sessions; healthy food workshops; attendance at School Nutrition Action Group/School Food Council meetings; and after school cooking, gardening and physical activities for parents and pupils to learn together. It was recommended that cooking clubs need to be delivered by a familiar member of staff, rather than somebody external, to appeal to parents. Improved communication about healthy lifestyle programmes between schools and parents (school website, newsletters, meetings), was also recommended.

## Discussion

Schools are appropriate places to promote physical activity and healthy eating because they can reach almost all children [40]. Our study explores school staff perceptions on the factors hindering and enabling successful implementation and sustainability of healthy eating and physical activity (healthy lifestyle) programmes, in the primary school setting.

A large sample of a diverse range of staff, were able to provide perspectives on factors relating to specific programmes and more generally, considering all healthy lifestyle programmes implemented at the school. The factors that were perceived to influence implementation of healthy lifestyle programmes, have been compared with the 23 factors described by Durlak and Dupre [26], in their framework of implementation factors. Many of the factors identified by staff in our study fit with the model, and suggest that these are important factors to consider when developing school-based healthy lifestyle interventions. Few of the factors highlighted in this study related directly to community level factors (outlined in the Durlak and Dupre framework [26]), such as politics and policy, perhaps because many of the interviews were with teaching staff. More interviews with school administrators or decision makers at school district level may have identified some broader community level factors as relevant. Most of the other factors identified aligned with the Durlak and Dupre model [26].

Unique findings from our study, not discussed previously in the Durlak and Dupre model [26], nor within any other implementation models reviewed [29–33], included the range of healthy lifestyle programmes delivered, timing for effective implementation and pupils leading on programme delivery. Despite complaints about an over-crowded curriculum, some schools delivered a number of programmes/initiatives with overlapping content. Head teachers therefore, need to be supported to identify and prioritise a few key evidence-based healthy lifestyle programmes or initiatives, with different key targets for behaviour change, so that teaching staff are not over-burdened. This is also a potential role for policy-makers. Timing of programme implementation, was also considered critical. It was important to staff to ensure that programmes are integrated into the curriculum plans (during their planning period), ready for the start of the following academic year. To facilitate this, teachers should be involved in the curriculum development for the programme, and implementation plan to ensure optimal implementation [41]. Pupils leading on aspects of programme delivery, was another important perceived facilitator in our study, not previously discussed in the implementation models reviewed [26, 29–33]. Previous research has suggested that children should be involved from the early stages when developing an intervention in order to make attractive interventions to pupils [41]. Programme developers and schools need to work together to plan how pupils can help lead on delivery of programme components.

The most critical factor perceived to hinder effective implementation in our study was limited time for delivery (particularly programme components not incorporated into the curriculum). Previous systematic reviews also reported time constraints as a critical barrier to effective implementation of physical activity [27] and health promotion programmes in schools [4, 7]. Greenberg et al., [33] also looked at implementation of school-based preventative programmes, and agreed with our findings, that lack of attention to the programme, due to competition in the curriculum, and lack of pre-planning time to look at programme resources, both hindered implementation. In order for schools to be able to prioritise healthy lifestyle education generally, and dedicate more time in the curriculum for its delivery, it is clear that the government needs to prioritise childhood obesity prevention and support primary schools to deliver more education around healthy eating and physical activity. There also needs to be more rigorous research conducted, to demonstrate causality between healthy eating and physical activity knowledge and academic achievement, a principal priority for schools (as there is some evidence to suggest association) [7]. Schools may be more willing and able to prioritise healthy lifestyle programmes, if it was clear that the aims of the programmes fit with school priorities for academic achievement.

The other factors identified as challenging implementation in our study, such as lack of training and support, insufficient resources, facilities and capacity (for cooking, healthy eating and physical activity programmes, after school mainly), align with previous reviews of implementation factors [26, 27, 30, 33]. Similar to previous reviews [26, 27, 30, 33], unsustainable funding and external support (for continued delivery of cooking and physical activity after school clubs), was also perceived to hinder implementation and sustainability in our study. Failing to see a need for the intervention (for example due to already established similar healthy lifestyle programmes/curriculum in place) and poor perceived skills proficiency (for cooking and physical education), were other perceived barriers also identified in previous literature [26, 30, 33]. Limited parent engagement and support was another important barrier, also identified previously [7, 27].

Important perceived enablers of implementation in our study, and consistent with previous reviews of implementation factors, were well-resourced programmes [27, 30, 31, 33], with accessible, adaptable, engaging, interactive, “hands-on” resources for cooking, gardening and physical activities (online format, age and culturally appropriate). Also congruent with previous reviews, were the following facilitators: good facilities and infrastructure [29, 33] (for cooking activities), staff availability [26, 30, 31] and support, with more volunteers to deliver programmes after school [7], and adequate funding [26, 31–33]. Integration of new programmes [26, 30, 31] into the curriculum, school structures and school food policies, was also thought to be an important enabler for implementation and sustainability. Staff perceptions of value of programme, commitment and buy-in also emerged as important factors in our study, and aligned with previous literature [26, 30, 31, 33]. Whole-school involvement and collaboration from all school partners, was considered equally important in our study for effective implementation, with strategies for building long-term capacity and support for programmes (particularly after school activities) recommended for effective sustainability. It was clear that capacity at school-level is a critical issue for effective implementation and sustainability of healthy lifestyle programmes. Programme providers need to work with the school senior leadership team to implement supportive strategies to enable school staff to deliver programmes long-term and ensure self-sufficiency in the provision of programmes in the long term. Using incentives or opportunities for Continued Professional Development could facilitate this. Furthermore, parents and the local community could also be supported to be more involved in delivery. Effective leadership (from head teacher and administrative team), was also a perceived enabler for implementation, along with a programme champion (for example a programme coordinator leading on programme delivery) to make it feasible at the school, and clear role delineation for staff delivering programmes. These factors have also been highlighted as essential components for implementation, in previous implementation literature [26, 30–32]. Also recommended in our study and consistent with previous reviews [26, 27, 29–31, 33], was effective training (delivered to all staff, interactive and practical), with adequate planning time, good communication and technical support from the programme team. Staff in our study also recommended that schools create more opportunities for parent involvement in healthy lifestyle programmes. Dimotrovich [31], supports that parents need to be specifically informed on the goals of the preventative interventions and involved in decision making. Staff recommended that pupils and parents learn about healthy lifestyle together, with after school clubs, events, school activities and homework including both parents and children. Previous research also supports that parents and children need to learn about healthy lifestyle together [4]. Parent governors could be consulted to devise other effective strategies for parent involvement.

The importance of adaptability (flexibility), with localised decision-making over delivery and good contextual fit, by tailoring the intervention to local need (timings, locations, resources, format) highlighted in our study, has also been identified as an important facilitating factor in the implementation literature previously [26, 27, 29–33]. Staff generally received guidance on programme delivery from the programme coordinator. Although giving individual staff autonomy and flexibility over programme delivery was seen as critical, this resulted in variability in programme delivery between schools and classes, and may compromise programme fidelity. Research suggests that modifications are necessary for successful implementation, because this improves fit between an intervention, its consumers and context and improves buy in [31]. However, negative adaptations, or lack of core components or poorly delivered core components, can hamper the impact of the intervention [31]. More research studies need to be conducted to inform the fidelity-adaptation debate, as we need to understand more fully, which intervention components can be modified, compared with those that need to be delivered exactly as they were developed and ways to make changes, whilst still achieving intended outcomes [33]. A component analysis can be carried out, to identify the core components vs. the modifiable components [29]. This is challenging, because they are often only identified through trial and error over time, and through more wide dissemination of the intervention over a variety of different contexts [29]. Assessments of the implementation quality of core elements, should therefore be used as process measures [31]. Once the critical core components or process elements are identified, the degree to which adaptation deviates from the model can be evaluated [31]. The programme team can then give more effective guidance to programme implementers concerning the core components of the programme to deliver with fidelity, and which components can be adapted and modified, so that implementers feel like there is some flexibility [29].

Our findings therefore, provide detailed contextual information relating to implementation and sustainability of healthy lifestyle programmes in the primary school setting. Many of the factors that emerged as important are congruent with the broader implementation literature [26, 29, 30, 32, 33]. Some of the factors influencing implementation identified in our study were not included as important constructs in any of the implementation models reviewed (timing of implementation, head teachers prioritising a few key healthy lifestyle programmes, pupils leading on aspects of programme delivery), or not included in all of the implementation models reviewed (parent involvement and time constraints). These factors should be given consideration by programme developers. This also strengthens the argument for a single multi-level ecological framework for understanding implementation [26] of school-based health promotion interventions (including healthy eating and physical activity programmes) [27], incorporating all of the key influencing factors identified from relevant research studies. Furthermore, there is convincing evidence of the need for comprehensive, whole-school, coordinated, multi-pronged health promotion strategies, incorporating both school level and teacher activities [27]. The challenge for future research is to address the barriers identified within current evidence, with evidence-based tailored implementation strategies, that allow for simple implementation and adaptation to specific settings, without compromising the core components of the intervention.

## Strengths and limitations

One strength of our study is that, unlike many other studies, it refers to a large amount of interview data containing the perspectives of a diverse range of primary school staff, as well as programme staff assigned to support schools with delivery. It presents insights from staff involved in the delivery of a large number of different healthy eating and physical activity behaviour change interventions, making the findings more generalisable to differing contexts. Unlike many other studies in this area, the study also presents recommended strategies for overcoming the barriers identified and specific recommendations for future healthy lifestyle programmes. The current study is limited, in that it only includes the perceptions of members of school staff and omits the experiences of pupils or parents who are involved in interventions. Their perspectives should also be considered, when developing health promotion programmes in the future. Researchers were already known to some head teachers through the recruitment process, and Year 2 and Year 4 teaching staff at the 8 schools involved in the PFS, through previous data collection with pupils. This may have resulted in some socially desirable responses. However, a vast amount of data was collected from staff not known to the research team, with different types of staff in agreement with the themes identified. Interviews were not fully transcribed. A process of ‘iterative listening’ was conducted, with only full transcription of key relevant sections. This methodology was perceived to be sufficient for the level of analysis needed for the evaluations. This may have limited the data made available for interpretation and analysis.

## Conclusions

Schools present an appropriate setting to promote healthy lifestyles and there is a need for easily implemented, tailored, evidence-based interventions designed to promote healthy lifestyles. Each intervention needs to fit well contextually into the school setting, to make implementation and sustainability effective. Programmes need to be integrated into the curriculum, school structures and school policies and aligned with curriculum objectives to be acceptable to staff. There needs to be effective support, commitment and leadership at multiple levels for success. Adequate training, resources and funding, right timing of implementation, as well as effective involvement of pupils and parents are also important factors. Head teachers need to be supported to be able to prioritise and commit to delivery of a few key healthy lifestyle programmes, in an overcrowded curriculum. The study provides recommendations for policy makers, programme providers and schools to inform the design, and implementation of future healthy lifestyle interventions in the school setting.

## Additional files

Additional file 1: Healthy lifestyle programmes/initiatives used by schools to target nutrition and physical activity behaviour change. Table of healthy lifestyle programmes or initiatives implemented at schools to target nutrition and physical activity behaviour change. (DOCX 17 kb)

Additional file 2: Interview topic guides for school and programme staff. Interview topic guides; questions for stakeholders. (DOCX 18 kb)

## Data Availability

The datasets used and/or analysed during the current study are available from the corresponding author on reasonable request.

## Abbreviations

CCG: Clinical Commissioning Group
FDE: Food Dudes Evaluation
HSC: Healthy School Coordinator
NCMP: National Child Measurement Programme
PE: Physical Education
PFS: PhunkyFoods Feasibility Study
PSHE: Personal, Social and Health Education
