# Supplementary material for: Effective implementation of primary school-based healthy lifestyle programmes: a qualitative study of views of school staff
Source: BMC Public Health. 2019 Sep 9;19:1239. doi: 10.1186/s12889-019-7550-2 (PMC6734437; doi:10.1186/s12889-019-7550-2)
Supplement: Supplementary file 1 — Healthy lifestyle programmes/initiatives used by schools to target nutrition and physical activity behaviour change. Table of healthy lifestyle programmes or initiatives implemented at schools to target nutrition and physical activity behaviour change. (DOCX 17 kb) [file 12889_2019_7550_MOESM1_ESM.docx]

*­*­­­­­­­­­Additional file 1. Healthy lifestyle programmes/initiatives used by schools to target nutrition and physical activity behaviour change

| **Programmes/ initiatives** | **Description of healthy lifestyle programmes/ initiatives** |
| --- | --- |
| PhunkyFoods programme | Online interactive cross-curricular healthy eating and physical activities lesson plans and resources (food models and food mats, food preparation resources, DVDs). A school curriculum that includes healthy eating, physical activity and body image. Increased sessions for physical activity (e.g. Science, Personal Social Health Education, Physical Education (PE) etc.), environments and cultural practices that support eating healthier foods and being active throughout each day (‘whole school approach’). A flexible approach to delivery e.g. breakfast, after-school or lunch club (PhunkyClub). Delivered as weekly sessions through embedding in the existing curriculum. Parent support and home activities that encourage children to be more active, eat more nutritious foods and spend less time in screen based activities. Regional community support workers offer ongoing training and support for teachers around the whole school approach to healthy lifestyles. |
| Food Dudes programme | Intervention based on positive role models (the Food Dudes characters), repeated tasting and rewards and incorporated three phases. Phase 1 (Intensive Phase) delivered in the classroom (20 days) involving repeated fruit and vegetable tasting with rewards for trying and fully consuming fruit and vegetables, watching Food Dudes DVDs/listening to emails, using snack boxes to bring in fruits and vegetables from home and recording of fruit and vegetable consumption at home for 28 days (home diary). Phase 2 (Dining Experience Phase) (4 weeks) involved pupils completing level cards to record and reward consumption of fruit and vegetables at lunchtime, behaviourally assisted replacement (BAR) techniques, whereby catering staff used behaviour change techniques such as prompting, presentation, branding, role modelling, rewards and competitions to encourage and facilitate healthy food choices. Pupils consumed entire portions of fruit and vegetables to receive ticks on their level cards; pupils received a reward for every level completed. Older pupils acted as Food Dude coaches to monitor fruit and vegetable consumption by ticking pupil’s level cards at lunchtime with assistance from the lunchtime supervisors. Phase 3 (Maintenance Phase - rest of school year) - schools encouraged to continue with the behaviour change techniques to maintain increased levels of fruit and vegetable consumption without the use of level cards or rewards and encouraged to embed the message in the school e.g. through the curriculum, events, Food Dudes conferences, school food ambassadors etc. Training and support provided by a behaviour change specialist. |
| Food for Life Partnership Award | School works towards an award to show how they embed food education into the curriculum, e.g. cooking activities – teach pupils cooking skills with use of vegetables from school gardens. Farm visits. Discourage unhealthy snacks, unhealthy lunchboxes. |
| Healthy Schools Award | Provision of healthy school meals, healthy lunchboxes and healthy snacks. Offers support with: Healthy Schools status (School Health Check); PSHE, school food, nutrition and cooking and physical activity etc. |
| ‘Bee Aware’ programme | - As part of government’s sustainable schools’ agenda - to reduce carbon emissions in schools by 2020. The programme encourages staff and pupils to think about issues such as climate change, pollution and global warming- makes schools environmentally aware. |
| Up Beat kids programme | A free community based weight management programme for young people aged 7-17 years old, who are overweight and their families. Sessions delivered after school for 10 weeks. |
| Tesco Farm to Fork project (Tesco Eat Happy Project) | Takes education out of the classroom and actively shows children where their food comes from. Visit suppliers at their farms or factories and gone on in-store Farm to Fork trails at Tesco stores provide practical demonstrations helping children explore fresh fruit and vegetables, taste cheeses, see how bread is baked and learn about the different species and origins of fish. |
| Fuel for School programme | Partners with local schools to provide intercepted food to feed their students with, and to help educate children about food and how to avoid waste. Enables schools to provide children and families with access to food on a Pay As You Feel basis (can be money or time/skills donation), schools receive deliveries of surplus food that would go to waste and offer market stalls to children and parents. Reduces the quantity of perfectly edible food that goes to waste; educates communities to make responsible choices about food for a sustainable future. Provides the opportunity to bridge the Holiday Hunger gap. Puts the United Nations Sustainable Development Goals at the heart of education. Provides training and educational resources covering key aspects of the national curriculum for school based staff. Providers deliver annual assemblies and workshops within schools. |
| Jamie Oliver Food Revolution Day | Kitchen Garden Project empowers primary school teachers to integrate growing and cooking into the school day. By teaching children about food, where it comes from, how to cook it and how it affects their bodies. Sell produce on market stalls for parents. |
| Cooking clubs (after school) | Learning how to cook and bake, basic food preparation skills. Some parent involvement at some schools. |
| Gardening initiatives/ clubs | Growing own produce in school garden, ground beds or nearby allotments to be used in school meals - cooking with produce. Some schemes in conjunction with Royal Horticultural Society and Santander. |
| Physical activity programmes / initiatives | Change 4 Life 10-week family exercise group – parents and children.  Running/walking a “Daily Mile” initiative.  Physical activity initiatives- Skip2bFit, ActivKids and the Bikeability scheme.  Coaches and Physical Education (PE) Specialists deliver PE curriculum.  Supervised games/physical activity sessions e.g. girls football, netball, cricket, organised games at lunchtimes run by external companies.  Wide range of after school sports clubs, e.g. football, rounders, gymnastics, karate, Judo, boxercise, dance etc.– delivered by external agencies, coaches etc.  Links with local high school and college for sport and PE delivery. Year 6 also act as Sports Leaders and help deliver sessions |
| School Food initiatives and policies | Breakfast clubs. Packed lunch policy. Menu changes. Healthier “grab bags” at lunchtimes (packed lunches provided by school). “Fruit Wednesdays” (provide only fruit for dessert). Changed ‘flight trays’ for plates and bowls in dining room. Creative fruit presentation (displays). Lunchtime supervisors to encourage children to eat healthily and check lunchboxes. More fruit provision - provision of fruit in a communal fruit bowl in the dining room for children with ‘unhealthy’ packed lunches.  Healthy lunchbox initiative- educate parents on what constitutes a healthy lunchbox with consultations and leaflets for parents.  Parents invited to dine with children at lunchtimes.  Healthy Tuck Shop.  Nutrition Action Group/ School Food Council - consult with pupils and parents to review school food culture. Role model healthy eating behaviours to their peers.  Use of food ambassadors – pupils assigned to promote healthy nutrition at school, support younger pupils with healthy eating behaviours and learning about healthy eating. Reward peers for healthy eating e.g. with stickers.  School shop selling school meals for the community. School meals for local ‘pensioners’. |
