# Supplementary material for: Effective implementation of primary school-based healthy lifestyle programmes: a qualitative study of views of school staff
Source: BMC Public Health. 2019 Sep 9;19:1239. doi: 10.1186/s12889-019-7550-2 (PMC6734437; doi:10.1186/s12889-019-7550-2)
Supplement: Supplementary file 2 — Interview topic guides for school and programme staff. Interview topic guides; questions for stakeholders. (DOCX 18 kb) [file 12889_2019_7550_MOESM2_ESM.docx]

Additional File 2. Interview topic guides for school and programme staff

| **Interview topic guide for school staff** |
| --- |
| ***Role***   - What is your responsibility within the school?   ***Types of healthy lifestyle initiatives/programmes***   - Are there any initiatives/programmes delivered in your school regarding healthy lifestyle (nutrition or physical activity)? *(E.g. PhunkyFoods, Food Dudes, MAF- Mission Active Future (school and community-based active play), Obesity Plan, Cookit, Change for Life. Kids on 10 week programme, cooking clubs, gardening clubs, sports clubs)* - If no, why is that?   ***Coordination and delivery***   - Who is involved in leading or co-ordinating delivery? - How are the programmes delivered? Through the curriculum or classroom activity? How many lessons? Which lessons? Through activities? Which activities? How many activities? - Which year groups have been involved and how often? Through breakfast clubs and/or after school clubs? - External Bodies? Other? - Are you able to comment on what pupils may have thought about these activities? Can you suggest any recommendations/improvements for the activities used? |
| ***Involvement and roles within healthy lifestyle programmes***   - What is your role within these programmes at the school? What are the roles of the catering team? What are the roles of the governors? Roles of teaching staff? Any other staff involved? Pupil’s involvement?   ***Parent involvement***   - What involvement do parents/carers have? *(E.g. PhunkyFoods Missions statements, Food Dudes snack boxes, after school clubs etc.)* If they are not involved, can you comment on why not? - How do you engage the above people and how well does this work? How do you think parents could be involved? |
| ***Training for programme delivery***   - Have those involved in promoting/delivering healthy lifestyle programmes attended or received training? Who received the training? What sort of training? - Who delivered it? When and where? How was it delivered? How many sessions and how long? - What did you/they think of the training? *(quantity, length, approach, training resources and delivery?)* Was anything missing from the training? Can you suggest any improvements or recommendations to the training? |
| ***Support***   - Have you required any support for these programmes? Were you able to access support if needed? If yes, from whom? And how often? Do you require any on-going support? - What did you think about the support received? Was anything missing? How would you want to be supported?   ***Curriculum on healthy lifestyle education***   - How is healthy lifestyle teaching (nutrition and physical activity) delivered at the school to pupils? Which lessons? How many lessons? Mode of delivery? What activities? How many activities? - Which staff are involved? (roles) Was there training provided to deliver teaching? (If haven’t discussed specific teacher training earlier) If yes, what sort of training? Who was it delivered by? When and where? Who received it? - What did you think of the training? (delivery, quality) Anything missing from the training?   ***Changes to the curriculum on healthy lifestyle education***   - Have there been any changes to teaching on diet and physical activity in the last year? *(prompt: in the whole school? In specific year groups?)* If yes, what has prompted these changes? - What have these changes involved? *(Prompt: any changes from addition of a programme? how is the programme used within the curriculum? e.g. replaced/ used additionally/ supplemented etc. What was the rationale behind using it this way?)*   ***Resources for programmes***   - What resources and activities are used in teaching/programmes? (*prompt: refer to specific programmes mentioned)* How are they obtained/accessed? On-line or hard copies e.g. manuals etc. - What do you think of the resources? Quality of materials? Suitability of materials? Quantity? Do you have any recommendations for the resources? Which resources do you use most often? Which ones do you not use/find useful? Which ones do the pupils like? And not like? Is anything missing? - Does the programme accommodate for different levels of ability? Is there enough work to do for those who may be more advanced? Is there enough support for those who may be struggling? |
| ***Effectiveness of programmes***   - Has this teaching (or programmes) had any effects on pupils’ (If yes please can they explain further?) Knowledge on healthy eating and physical activity? Attitudes towards healthy eating and physical activity? Eating and physical activity behaviours? Academic performance – engagement with the resources and activities from healthy eating and physical activity teaching? Attendance and behaviour – if they can comment on them? Social interaction? Anything else? - Any effects on parents of pupils? (More involvement at school? How they take children to school?) Any effects on Staff? |
| ***Challenges/barriers to success***   - Have there been any challenges/barriers to incorporating healthy lifestyle programmes or initiatives into the school? If yes, please explain *(prompt: refer to specific programmes e.g. challenges/barriers in delivering any new teaching on diet and physical activity? Specific programme activities?*) - What would help to prevent these challenges in the future? |
| ***Key learning, recommendations and future plans***   - What do you think has worked well about current healthy lifestyle programmes at the school? Or current nutrition and physical activity learning? *(prompt: teaching materials, resources/ activities, delivery?)* - Is there anything that has not worked well? - Do you think these programmes are sustainable? Will you continue to use the programmes next year? What are your future plans for these programmes? For healthy lifestyle teaching? - Will you use existing resources?   *(Prompt: refer to specific programmes mentioned e.g. how complete do you think the programme is? Is anything missing? Anything you would want to add or change?)*   - Can you suggest any improvements or recommendations to these programmes? Can you suggest any improvements or recommendations to teaching? Training materials and teaching resources? - What key elements would you want to see in any healthy lifestyle programmes based at the school? - What might make them effective or sustainable? - Are there any future plans for other healthy lifestyle programmes to be implemented at the school? |
| **Interview topic guide for programme staff supporting schools with delivery of programme**  ***Role***   - Please can you briefly describe what your role entails? for example, in a typical week? *(Brief description of activities, community initiatives work?)* - Have you received any training for this role? Would any specific training be useful? How long have you had this role? How and why was this role established? How long do you intend to keep working in this role?   ***Support for schools***   - How many of the schools are you currently working with/supporting? How have you supported each school? *(Visits/emails/phone calls)* How often have you visited each school? Who did you organise these visits with? *(Coordinator/other staff?)* What kind of support have they required? - Which members of staff have you supported? (*What was your experience of supporting the different types of staff? What were their different needs? E.g. needs of different Year groups?)* Have you been able to meet all their support needs? Was there anything you could not help them with? - Have there been any barriers to providing support? *(communication, time, budget)*   ***Training for schools***  Have you been involved in any training of school staff for delivery of the programme? *(Where? How? To which members of staff?)* How many schools and how many times? How was this training delivered?   - Were there any barriers or challenges to delivering the training? Can you suggest any recommendations or improvements for training?   ***Impacts***   - Have there been any impacts on the schools from your support for the programme? *(Examples of activities established)* - As a result of your support or involvement did any activities/initiatives arise that you had not expected? *(e.g. any other activities established? e.g. clubs replaced with programme activities)*   ***Parent engagement***   - Are parents involved in the programme at all? How are they involved? *(Activities/homework/clubs)* - Have schools requested any support for parent engagement? If so, how have you supported them?   ***Future plans***   - Have the schools discussed their future plans for the programme with you? Will they require any further/sustained support? If yes, what will this involve? |
